# Di-μ-sulfido-bis­{[*rac*-1,2-bis­(η^5^-4,5,6,7-tetra­hydro­inden-1-yl)ethane]­zirconium(IV)} toluene monosolvate

**DOI:** 10.1107/S1600536812044121

**Published:** 2012-10-31

**Authors:** Martin Haehnel, Kai Altenburger, Anke Spannenberg, Perdita Arndt, Uwe Rosenthal

**Affiliations:** a Leibniz-Institut für Katalyse e. V. an der Universität Rostock, Albert-Einstein-Strasse 29a, 18059 Rostock, Germany

## Abstract

The title dimeric zirconium complex, [Zr_2_(C_20_H_24_)_2_S_2_]·C_7_H_8_, was obtained from the reaction of (ebthi)Zr(η^2^-Me_3_Si–C_2_–SiMe_3_) [ebthi is *rac*-1,2-bis­(η^5^-4,5,6,7-tetra­hydro­inden-1-yl)ethane] and S=C=N-ada (ada = adamantan-1-yl) along with the formation of the isonitrile C N-ada. Each Zr^IV^ atom is coordinated by the sterically hindered ebthi ligand and two μ-sulfide ligands in a strongly distorted tetra­hedral geometry. The [ZrS]_2_ unit is almost planar (mean deviation from the best plane of the four atoms = 0.025 Å). A –CH_2_—CH_2_– group in one ebthi ligand was disordered over two sites, with refined occupancy factors of 0.551 (6) and 0.449 (6). The asymmetric unit also contains a toluene solvent mol­ecule.

## Related literature

For μ-sulfide-bridged [Cp_2_ *M*(μ-S)]_2_ metallocenes (Cp = C_5_H_5_), see: for *M* = Zr, Bottomley *et al.* (1986 ▶); Hey *et al.* (1987 ▶); for *M* = Nb, Skripkin *et al.* (1984 ▶). Furthermore, for [Cp′_2_Th(μ-S)]_2_ (Cp′ = 1,2,4-tri-*tert*-butyl­cyclo­penta­dien­yl), see: Ren *et al.* (2011 ▶); for [Cp′_2_Ta(μ-S)]_2_ (Cp′ = C_5_H_4_Me), see: Winkler *et al.* (1998 ▶). The starting alkyne complex (ebthi)Zr(η^2^-Me_3_Si–C_2_–SiMe_3_) was described by Lefeber *et al.* (1996 ▶). For μ-sulfide complexes with a further bridged ligand Cp′*M*(μ-S)_2_ *LM*Cp′, *L* = μ_2_-η^10^-fulvalene, Cp′ = Cp, *M* = Zr, see: Wielstra *et al.* (1990 ▶), *L* = μ_2_-bis­(η^5^-cyclo­penta­dien­yl)dimethyl­silane, Cp′ = Cp, *M* = Zr, see: Cacciola *et al.* (1992 ▶), *L* = μ_2_-bis­(η^5^-cyclo­penta­dien­yl)dimethyl­silane, Cp′ = Cp* (C_5_Me_5_), *M* = Zr, see: Burstynowicz & Petersen (1995 ▶).
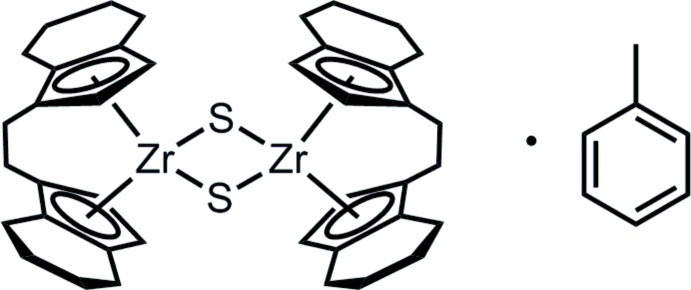


## Experimental

### 

#### Crystal data

[Zr_2_(C_20_H_24_)_2_S_2_]·C_7_H_8_
*M* *_r_* = 867.48
Monoclinic, 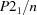

*a* = 12.6436 (3) Å
*b* = 20.9735 (4) Å
*c* = 14.8855 (3) Å
β = 105.092 (2)°
*V* = 3811.20 (14) Å^3^
*Z* = 4
Mo *K*α radiation
μ = 0.69 mm^−1^
*T* = 150 K
0.60 × 0.30 × 0.25 mm

#### Data collection

Stoe IPDS II diffractometer
Absorption correction: numerical (*X-SHAPE* and *X-RED32*; Stoe & Cie, 2005 ▶) *T*_min_ = 0.777, *T*_max_ = 0.854
65438 measured reflections
9109 independent reflections
7869 reflections with *I* > 2σ(*I*)
*R*_int_ = 0.036

#### Refinement

*R*[*F* ^2^ > 2σ(*F* ^2^)] = 0.027
*wR*(*F* ^2^) = 0.072
*S* = 1.05
9109 reflections
460 parameters
15 restraints
H-atom parameters constrained
Δρ_max_ = 1.30 e Å^−3^
Δρ_min_ = −0.89 e Å^−3^

### 

Data collection: *X-AREA* (Stoe & Cie, 2005 ▶); cell refinement: *X-AREA*; data reduction: *X-AREA*; program(s) used to solve structure: *SHELXS97* (Sheldrick, 2008 ▶); program(s) used to refine structure: *SHELXL97* (Sheldrick, 2008 ▶); molecular graphics: *XP* in *SHELXTL* (Sheldrick, 2008 ▶); software used to prepare material for publication: *SHELXL97*.

## Supplementary Material

Crystal structure: contains datablock(s) I, global. DOI: 10.1107/S1600536812044121/pk2450sup1.cif

Structure factors: contains datablock(s) I. DOI: 10.1107/S1600536812044121/pk2450Isup2.hkl

Additional supplementary materials:  crystallographic information; 3D view; checkCIF report

## Figures and Tables

**Fig. 1.**
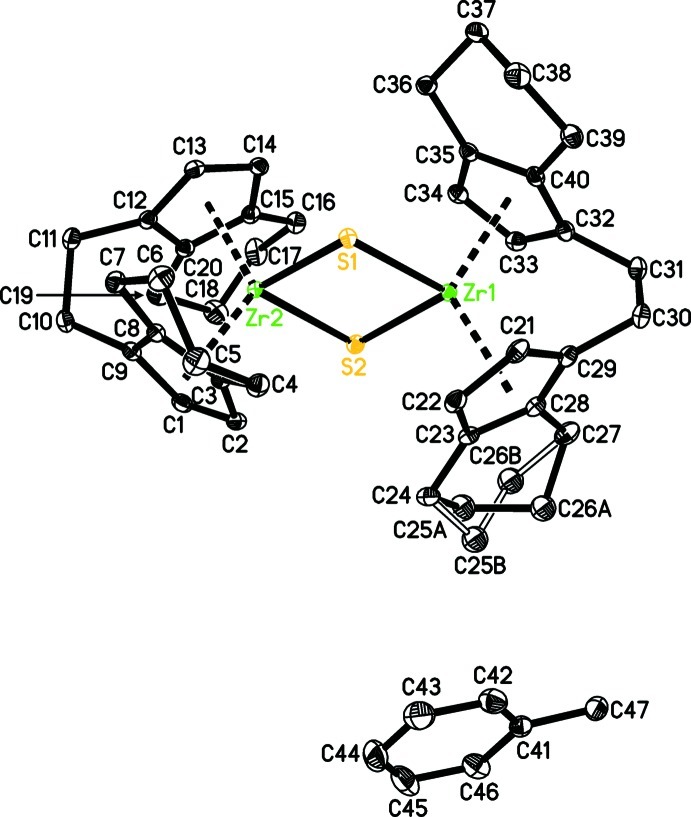
The molecular structure of the title compound with 30% displacement ellipsoids. Hydrogen atoms are omitted for clarity. Minor disorder is indicated by open bonds.

## supplementary crystallographic information

### Comment

The reaction of isothiocyanates with metallocene precursors *L*_2_M(η^2^-Me_3_Si—C_2_—SiMe_3_) (*L*_2_ = ebthi, Cp_2_, Cp*_2_, *M* = Ti, Zr) was investigated with the aim of synthesizing new 4-membered hetero-metallacycles. In this reaction, a C—S bond cleavage occurred leading to two different products. In addition to a dimeric bis(µ-sulfido) bridged zirconium complex, the formation of the organic *N-*adamantyl-isocyanide was observed.

In the title compound each zirconium atom is coordinated by two sulfido and the chelating ebthi ligand (Fig. 1). The geometry at the zirconium centers is strongly distorted tetrahedral. The largest deviation from the ideal tetrahedral angle is observed for S1—Zr2—S2 angle with 89.27 (2)°. The [ZrS]_2_ unit is almost planar (mean deviation from the best plane: 0.025 Å); bond lengths and angles of the [ZrS]_2_ unit are comparable with those of [Cp_2_Zr(µ-S)]_2_ (Bottomley *et al.*, 1986 and Hey *et al.*, 1987). Angles between the planes defined by Zr1, S1, Zr2, S2 and each of the five-membered rings of the ebthi ligands are 31.67 (8)° and 28.28 (5)° (for the ligand coordinated to Zr2), 30.74 (5)° and 28.86 (9)° (for the ligand coordinated to Zr1), respectively. The asymmetric unit of the title compound also contains a toluene solvent molecule.

### Experimental

To a solution of 469 mg (0,892 mmol) of (ebthi)Zr(η^2^-Me_3_Si—C_2_—SiMe_3_) in 15 ml of *n*-hexane was added dropwise a solution of 172 mg (0,892 mmol) *N*-adamantylisothiocyanate in 10 ml of *n*-hexane. Instantly, the reaction mixture turned from green to dark red and a colourless solid precipitated. The reaction mixture was allowed to stand for 12 h. After filtration, the dark red solution was evaporated *in vacuo*. The precipitate was dissolved in 8 ml of toluene, filtered and stored at -40°C. After 7 days, the dark red crystalline product was filtered, washed with cold toluene and dried *in vacuo*. Yield: 79% (273 mg, 0.212 mmol). Crystals suitable for X-ray analysis were obtained from a saturated solution in toluene at -40°C.

### Refinement

H atoms were placed in idealized positions with d(C—H) = 0.95 Å (CH), 0.99 Å (CH_2_) and 0.98 Å (CH_3_) and refined using a riding model with *U*_iso_(H) fixed at 1.2 *U*_eq_(C) for CH, CH_2_ and 1.5 *U*_eq_(C) for CH_3_. Atoms C25A, C26A and C25B, C26B are disordered over two sites with occupancies of 0.551 (6):0.449 (6). The SADI instruction was used to improve the geometry of this disordered part of the complex molecule. The largest diff. peak/hole (1.30/-0.89 e Å^-3^) were 0.58 Å from C26B and 0.36 Å from C25B, respectively. Additional difference map peaks of 0.97 and 0.89 e Å^-3^ were consistent with disorder of atoms C17 and C18, but was not modelled owing to the low occupancy and comparatively poor definition of the minor component.

### Crystal data

**Table:**

[Zr_2_(C_20_H_24_)_2_S_2_]·C_7_H_8_	*F*(000) = 1800
*M**_r_* = 867.48	*D*_x_ = 1.512 Mg m^−^^3^
Monoclinic, *P*2_1_/*n*	Mo *K*α radiation, λ = 0.71073 Å
*a* = 12.6436 (3) Å	Cell parameters from 15221 reflections
*b* = 20.9735 (4) Å	θ = 1.7–28.4°
*c* = 14.8855 (3) Å	µ = 0.69 mm^−^^1^
β = 105.092 (2)°	*T* = 150 K
*V* = 3811.20 (14) Å^3^	Prism, red
*Z* = 4	0.60 × 0.30 × 0.25 mm

### Data collection

**Table:**

Stoe IPDS II diffractometer	9109 independent reflections
Radiation source: fine-focus sealed tube	7869 reflections with *I* > 2σ(*I*)
Graphite monochromator	*R*_int_ = 0.036
ω scans	θ_max_ = 27.9°, θ_min_ = 1.7°
Absorption correction: numerical (*X-SHAPE* and *X-RED32*; Stoe & Cie, 2005)	*h* = −16→16
*T*_min_ = 0.777, *T*_max_ = 0.854	*k* = −27→27
65438 measured reflections	*l* = −19→19

### Refinement

**Table:**

Refinement on *F*^2^	Primary atom site location: structure-invariant direct methods
Least-squares matrix: full	Secondary atom site location: difference Fourier map
*R*[*F*^2^ > 2σ(*F*^2^)] = 0.027	Hydrogen site location: inferred from neighbouring sites
*wR*(*F*^2^) = 0.072	H-atom parameters constrained
*S* = 1.05	*w* = 1/[σ^2^(*F*_o_^2^) + (0.045*P*)^2^ + 1.0943*P*] where *P* = (*F*_o_^2^ + 2*F*_c_^2^)/3
9109 reflections	(Δ/σ)_max_ = 0.001
460 parameters	Δρ_max_ = 1.30 e Å^−^^3^
15 restraints	Δρ_min_ = −0.89 e Å^−^^3^

### Special details

**Table:**

Geometry. All e.s.d.'s (except the e.s.d. in the dihedral angle between two l.s. planes) are estimated using the full covariance matrix. The cell e.s.d.'s are taken into account individually in the estimation of e.s.d.'s in distances, angles and torsion angles; correlations between e.s.d.'s in cell parameters are only used when they are defined by crystal symmetry. An approximate (isotropic) treatment of cell e.s.d.'s is used for estimating e.s.d.'s involving l.s. planes.
Refinement. Refinement of *F*^2^ against ALL reflections. The weighted *R*-factor *wR* and goodness of fit *S* are based on *F*^2^, conventional *R*-factors *R* are based on *F*, with *F* set to zero for negative *F*^2^. The threshold expression of *F*^2^ > σ(*F*^2^) is used only for calculating *R*-factors(gt) *etc*. and is not relevant to the choice of reflections for refinement. *R*-factors based on *F*^2^ are statistically about twice as large as those based on *F*, and *R*- factors based on ALL data will be even larger.

### Fractional atomic coordinates and isotropic or equivalent isotropic displacement parameters (Å^2^)

**Table:**

	*x*	*y*	*z*	*U*_iso_*/*U*_eq_	Occ. (<1)
C25A	0.2121 (3)	0.1623 (2)	0.4520 (3)	0.0404 (12)*	0.551 (6)
H25A	0.2280	0.2037	0.4848	0.049*	0.551 (6)
H25B	0.1732	0.1717	0.3866	0.049*	0.551 (6)
C26A	0.1337 (4)	0.12520 (15)	0.4955 (3)	0.0382 (12)*	0.551 (6)
H26A	0.1699	0.1137	0.5608	0.046*	0.551 (6)
H26B	0.0676	0.1508	0.4943	0.046*	0.551 (6)
C25B	0.2419 (3)	0.1579 (3)	0.5029 (3)	0.0382 (14)*	0.449 (6)
H25C	0.2474	0.2049	0.5075	0.046*	0.449 (6)
H25D	0.2546	0.1397	0.5661	0.046*	0.449 (6)
C26B	0.1309 (4)	0.13715 (14)	0.4406 (4)	0.0396 (15)*	0.449 (6)
H26C	0.1264	0.1520	0.3766	0.048*	0.449 (6)
H26D	0.0731	0.1598	0.4618	0.048*	0.449 (6)
C1	0.50936 (16)	0.19785 (9)	0.22541 (14)	0.0230 (4)	
H1	0.4687	0.2364	0.2199	0.028*	
C2	0.52098 (16)	0.15239 (9)	0.29785 (13)	0.0225 (4)	
H2	0.4900	0.1554	0.3494	0.027*	
C3	0.58632 (15)	0.10219 (9)	0.28007 (12)	0.0202 (4)	
C4	0.63471 (16)	0.04717 (10)	0.34053 (13)	0.0250 (4)	
H4A	0.5827	0.0110	0.3288	0.030*	
H4B	0.6486	0.0595	0.4068	0.030*	
C5	0.74167 (18)	0.02721 (11)	0.31970 (15)	0.0310 (4)	
H5A	0.7700	−0.0119	0.3551	0.037*	
H5B	0.7969	0.0613	0.3395	0.037*	
C6	0.72301 (18)	0.01473 (10)	0.21574 (15)	0.0286 (4)	
H6A	0.6648	−0.0178	0.1958	0.034*	
H6B	0.7911	−0.0026	0.2041	0.034*	
C7	0.68978 (16)	0.07526 (9)	0.15759 (13)	0.0224 (4)	
H7A	0.7562	0.1004	0.1579	0.027*	
H7B	0.6543	0.0632	0.0924	0.027*	
C8	0.61232 (15)	0.11573 (9)	0.19452 (13)	0.0190 (3)	
C9	0.56845 (15)	0.17622 (9)	0.16281 (13)	0.0207 (4)	
C10	0.58321 (16)	0.21113 (9)	0.07888 (13)	0.0237 (4)	
H10A	0.6624	0.2165	0.0844	0.028*	
H10B	0.5503	0.2541	0.0767	0.028*	
C11	0.53012 (16)	0.17568 (9)	−0.01194 (13)	0.0238 (4)	
H11A	0.5184	0.2058	−0.0649	0.029*	
H11B	0.5800	0.1416	−0.0220	0.029*	
C12	0.42250 (15)	0.14693 (9)	−0.00862 (12)	0.0198 (3)	
C13	0.39478 (16)	0.08163 (9)	−0.01703 (12)	0.0199 (3)	
H13	0.4421	0.0479	−0.0241	0.024*	
C14	0.28447 (16)	0.07491 (9)	−0.01315 (12)	0.0204 (3)	
H14	0.2451	0.0360	−0.0173	0.025*	
C15	0.24360 (15)	0.13585 (9)	−0.00212 (12)	0.0202 (3)	
C16	0.12816 (16)	0.15500 (10)	−0.00663 (14)	0.0245 (4)	
H16A	0.1131	0.1472	0.0545	0.029*	
H16B	0.0769	0.1286	−0.0536	0.029*	
C17	0.10925 (19)	0.22487 (10)	−0.03208 (18)	0.0351 (5)	
H17A	0.1057	0.2307	−0.0988	0.042*	
H17B	0.0382	0.2385	−0.0221	0.042*	
C18	0.19963 (19)	0.26570 (11)	0.02570 (18)	0.0361 (5)	
H18A	0.2048	0.2584	0.0924	0.043*	
H18B	0.1813	0.3112	0.0120	0.043*	
C19	0.31256 (18)	0.25125 (9)	0.00682 (15)	0.0267 (4)	
H19A	0.3155	0.2705	−0.0532	0.032*	
H19B	0.3717	0.2704	0.0565	0.032*	
C20	0.32987 (16)	0.18022 (9)	0.00388 (12)	0.0198 (3)	
C21	0.32446 (17)	−0.04410 (10)	0.40258 (13)	0.0247 (4)	
H21	0.3561	−0.0838	0.3931	0.030*	
C22	0.37861 (17)	0.01546 (10)	0.41415 (13)	0.0257 (4)	
H22	0.4526	0.0227	0.4137	0.031*	
C23	0.30386 (17)	0.06192 (10)	0.42636 (13)	0.0243 (4)	
C24	0.3222 (2)	0.13084 (11)	0.45239 (15)	0.0367 (5)	
H24A	0.3552	0.1528	0.4075	0.044*	0.551 (6)
H24B	0.3733	0.1345	0.5151	0.044*	0.551 (6)
H24C	0.3168	0.1560	0.3951	0.044*	0.449 (6)
H24D	0.3975	0.1360	0.4926	0.044*	0.449 (6)
C27	0.10359 (19)	0.06559 (10)	0.43584 (15)	0.0321 (5)	
H27A	0.0630	0.0361	0.4666	0.038*	0.551 (6)
H27B	0.0542	0.0777	0.3750	0.038*	0.551 (6)
H27C	0.0891	0.0512	0.4949	0.038*	0.449 (6)
H27D	0.0378	0.0569	0.3844	0.038*	0.449 (6)
C28	0.20182 (16)	0.03123 (9)	0.41942 (13)	0.0222 (4)	
C29	0.21537 (16)	−0.03484 (9)	0.40748 (13)	0.0221 (4)	
C30	0.12928 (18)	−0.08483 (10)	0.40185 (15)	0.0291 (4)	
H30A	0.1059	−0.0849	0.4604	0.035*	
H30B	0.1606	−0.1273	0.3949	0.035*	
C31	0.02935 (17)	−0.07298 (10)	0.31952 (15)	0.0287 (4)	
H31A	−0.0139	−0.1127	0.3049	0.034*	
H31B	−0.0178	−0.0400	0.3368	0.034*	
C32	0.06350 (16)	−0.05142 (9)	0.23489 (13)	0.0224 (4)	
C33	0.03826 (15)	0.00736 (9)	0.18818 (14)	0.0225 (4)	
H33	−0.0015	0.0413	0.2061	0.027*	
C34	0.08243 (15)	0.00712 (9)	0.10987 (13)	0.0208 (4)	
H34	0.0773	0.0408	0.0664	0.025*	
C35	0.13508 (15)	−0.05170 (9)	0.10778 (13)	0.0195 (3)	
C36	0.18216 (16)	−0.07917 (9)	0.03361 (13)	0.0226 (4)	
H36A	0.2589	−0.0648	0.0435	0.027*	
H36B	0.1398	−0.0638	−0.0281	0.027*	
C37	0.17838 (18)	−0.15160 (10)	0.03607 (15)	0.0284 (4)	
H37A	0.1014	−0.1662	0.0156	0.034*	
H37B	0.2183	−0.1693	−0.0073	0.034*	
C38	0.2300 (2)	−0.17573 (10)	0.13383 (16)	0.0327 (5)	
H38A	0.3054	−0.1588	0.1555	0.039*	
H38B	0.2346	−0.2228	0.1328	0.039*	
C39	0.16376 (18)	−0.15550 (9)	0.20201 (15)	0.0279 (4)	
H39A	0.0989	−0.1835	0.1938	0.033*	
H39B	0.2094	−0.1608	0.2666	0.033*	
C40	0.12679 (15)	−0.08734 (9)	0.18699 (13)	0.0207 (3)	
C41	0.33335 (17)	0.19999 (10)	0.74341 (14)	0.0279 (4)	
C42	0.4157 (2)	0.16460 (13)	0.72132 (17)	0.0386 (5)	
H42	0.4100	0.1195	0.7181	0.046*	
C43	0.5059 (2)	0.19406 (17)	0.7039 (2)	0.0524 (7)	
H43	0.5611	0.1691	0.6879	0.063*	
C44	0.5164 (2)	0.25879 (17)	0.7095 (2)	0.0562 (8)	
H44	0.5785	0.2789	0.6974	0.067*	
C45	0.4366 (3)	0.29465 (14)	0.7328 (2)	0.0530 (7)	
H45	0.4441	0.3397	0.7374	0.064*	
C46	0.3448 (2)	0.26551 (11)	0.74982 (17)	0.0373 (5)	
H46	0.2900	0.2907	0.7658	0.045*	
C47	0.23410 (19)	0.16771 (12)	0.75869 (16)	0.0353 (5)	
H47A	0.2554	0.1405	0.8140	0.053*	
H47B	0.2001	0.1415	0.7043	0.053*	
H47C	0.1817	0.1999	0.7678	0.053*	
S1	0.39577 (4)	−0.00729 (2)	0.19018 (3)	0.01783 (9)	
S2	0.24917 (4)	0.12761 (2)	0.22003 (3)	0.01908 (9)	
Zr1	0.239232 (13)	0.013567 (8)	0.256989 (11)	0.01540 (5)	
Zr2	0.400064 (14)	0.106505 (8)	0.147091 (11)	0.01518 (5)	

### Atomic displacement parameters (Å^2^)

**Table:**

	*U*^11^	*U*^22^	*U*^33^	*U*^12^	*U*^13^	*U*^23^
C1	0.0213 (9)	0.0203 (8)	0.0276 (9)	−0.0034 (7)	0.0067 (7)	−0.0073 (7)
C2	0.0213 (9)	0.0272 (9)	0.0198 (8)	−0.0051 (7)	0.0067 (7)	−0.0069 (7)
C3	0.0182 (9)	0.0257 (9)	0.0160 (8)	−0.0038 (7)	0.0035 (7)	−0.0030 (7)
C4	0.0221 (9)	0.0334 (10)	0.0176 (8)	−0.0009 (8)	0.0017 (7)	0.0032 (8)
C5	0.0238 (10)	0.0404 (12)	0.0263 (10)	0.0041 (9)	0.0021 (8)	0.0046 (9)
C6	0.0273 (10)	0.0309 (10)	0.0279 (10)	0.0062 (8)	0.0077 (8)	0.0009 (8)
C7	0.0205 (9)	0.0255 (9)	0.0226 (9)	−0.0003 (7)	0.0080 (7)	−0.0007 (7)
C8	0.0164 (8)	0.0218 (8)	0.0186 (8)	−0.0037 (6)	0.0045 (7)	−0.0026 (7)
C9	0.0194 (9)	0.0199 (8)	0.0225 (9)	−0.0041 (7)	0.0052 (7)	−0.0033 (7)
C10	0.0223 (9)	0.0231 (9)	0.0268 (9)	−0.0047 (7)	0.0082 (8)	0.0019 (7)
C11	0.0245 (10)	0.0261 (9)	0.0227 (9)	−0.0017 (7)	0.0096 (7)	0.0034 (7)
C12	0.0234 (9)	0.0206 (8)	0.0155 (8)	−0.0001 (7)	0.0051 (7)	0.0018 (6)
C13	0.0245 (9)	0.0190 (8)	0.0161 (8)	0.0020 (7)	0.0050 (7)	−0.0007 (7)
C14	0.0246 (9)	0.0191 (8)	0.0169 (8)	−0.0022 (7)	0.0042 (7)	0.0003 (7)
C15	0.0212 (9)	0.0224 (9)	0.0161 (8)	0.0007 (7)	0.0030 (7)	0.0027 (7)
C16	0.0196 (9)	0.0282 (9)	0.0245 (9)	0.0020 (7)	0.0034 (7)	0.0041 (8)
C17	0.0293 (11)	0.0288 (10)	0.0476 (13)	0.0062 (9)	0.0107 (10)	0.0095 (10)
C18	0.0331 (12)	0.0261 (10)	0.0484 (14)	0.0049 (9)	0.0092 (10)	−0.0019 (9)
C19	0.0316 (11)	0.0174 (8)	0.0293 (10)	0.0015 (8)	0.0050 (8)	0.0008 (7)
C20	0.0229 (9)	0.0196 (8)	0.0164 (8)	0.0005 (7)	0.0042 (7)	0.0012 (6)
C21	0.0264 (10)	0.0300 (10)	0.0181 (8)	0.0077 (8)	0.0065 (7)	0.0063 (7)
C22	0.0198 (9)	0.0392 (11)	0.0167 (8)	−0.0012 (8)	0.0023 (7)	0.0029 (8)
C23	0.0301 (10)	0.0283 (10)	0.0141 (8)	−0.0036 (8)	0.0050 (7)	−0.0008 (7)
C24	0.0554 (15)	0.0327 (11)	0.0228 (10)	−0.0155 (10)	0.0119 (10)	−0.0081 (9)
C27	0.0333 (11)	0.0382 (11)	0.0277 (10)	0.0095 (9)	0.0134 (9)	−0.0009 (9)
C28	0.0247 (9)	0.0264 (9)	0.0165 (8)	0.0025 (7)	0.0071 (7)	0.0016 (7)
C29	0.0250 (10)	0.0249 (9)	0.0171 (8)	0.0010 (7)	0.0067 (7)	0.0043 (7)
C30	0.0352 (11)	0.0286 (10)	0.0269 (10)	−0.0061 (9)	0.0142 (9)	0.0051 (8)
C31	0.0256 (10)	0.0333 (11)	0.0312 (10)	−0.0083 (8)	0.0145 (8)	−0.0016 (8)
C32	0.0174 (9)	0.0254 (9)	0.0248 (9)	−0.0037 (7)	0.0059 (7)	−0.0018 (7)
C33	0.0172 (9)	0.0248 (9)	0.0243 (9)	0.0005 (7)	0.0035 (7)	−0.0034 (7)
C34	0.0182 (9)	0.0207 (8)	0.0206 (8)	0.0000 (7)	0.0000 (7)	0.0002 (7)
C35	0.0160 (8)	0.0207 (8)	0.0200 (8)	−0.0031 (6)	0.0017 (7)	−0.0016 (7)
C36	0.0209 (9)	0.0267 (9)	0.0196 (8)	−0.0022 (7)	0.0040 (7)	−0.0034 (7)
C37	0.0307 (11)	0.0247 (9)	0.0326 (10)	−0.0046 (8)	0.0134 (9)	−0.0089 (8)
C38	0.0362 (12)	0.0222 (9)	0.0417 (12)	0.0058 (8)	0.0134 (10)	0.0003 (9)
C39	0.0335 (11)	0.0201 (9)	0.0304 (10)	−0.0007 (8)	0.0087 (9)	0.0022 (8)
C40	0.0178 (8)	0.0209 (8)	0.0230 (9)	−0.0040 (7)	0.0044 (7)	−0.0008 (7)
C41	0.0255 (10)	0.0341 (11)	0.0212 (9)	−0.0011 (8)	0.0009 (7)	0.0035 (8)
C42	0.0326 (12)	0.0429 (13)	0.0388 (12)	−0.0003 (10)	0.0068 (10)	−0.0076 (10)
C43	0.0313 (13)	0.079 (2)	0.0486 (15)	−0.0056 (13)	0.0139 (12)	−0.0165 (15)
C44	0.0338 (14)	0.083 (2)	0.0502 (16)	−0.0235 (14)	0.0073 (12)	0.0054 (15)
C45	0.0510 (17)	0.0402 (14)	0.0564 (17)	−0.0174 (12)	−0.0062 (13)	0.0109 (12)
C46	0.0334 (12)	0.0328 (11)	0.0392 (12)	0.0031 (9)	−0.0021 (10)	0.0032 (9)
C47	0.0323 (12)	0.0446 (13)	0.0289 (11)	−0.0055 (10)	0.0080 (9)	0.0020 (9)
S1	0.0170 (2)	0.01675 (19)	0.0200 (2)	0.00062 (15)	0.00529 (16)	0.00071 (15)
S2	0.0206 (2)	0.0177 (2)	0.0203 (2)	0.00193 (16)	0.00786 (17)	0.00083 (16)
Zr1	0.01483 (9)	0.01683 (8)	0.01446 (8)	−0.00059 (6)	0.00366 (6)	0.00105 (6)
Zr2	0.01572 (9)	0.01542 (8)	0.01456 (8)	−0.00112 (6)	0.00423 (6)	−0.00050 (6)

### Geometric parameters (Å, º)

**Table:**

C25A—C26A	1.531 (2)	C21—C29	1.413 (3)
C25A—C24	1.539 (2)	C21—Zr1	2.4707 (19)
C25A—H25A	0.9900	C21—H21	0.9500
C25A—H25B	0.9900	C22—C23	1.402 (3)
C26A—C27	1.523 (2)	C22—Zr1	2.5363 (19)
C26A—H26A	0.9900	C22—H22	0.9500
C26A—H26B	0.9900	C23—C28	1.421 (3)
C25B—C24	1.522 (2)	C23—C24	1.499 (3)
C25B—C26B	1.531 (2)	C23—Zr1	2.6419 (18)
C25B—H25C	0.9900	C24—H24A	0.9900
C25B—H25D	0.9900	C24—H24B	0.9900
C26B—C27	1.537 (2)	C24—H24C	0.9900
C26B—H26C	0.9900	C24—H24D	0.9900
C26B—H26D	0.9900	C27—C28	1.510 (3)
C1—C9	1.412 (3)	C27—H27A	0.9900
C1—C2	1.418 (3)	C27—H27B	0.9900
C1—Zr2	2.4699 (18)	C27—H27C	0.9900
C1—H1	0.9500	C27—H27D	0.9900
C2—C3	1.405 (3)	C28—C29	1.413 (3)
C2—Zr2	2.5521 (18)	C28—Zr1	2.6081 (18)
C2—H2	0.9500	C29—C30	1.498 (3)
C3—C8	1.424 (2)	C29—Zr1	2.5479 (18)
C3—C4	1.493 (3)	C30—C31	1.534 (3)
C3—Zr2	2.6542 (18)	C30—H30A	0.9900
C4—C5	1.523 (3)	C30—H30B	0.9900
C4—H4A	0.9900	C31—C32	1.504 (3)
C4—H4B	0.9900	C31—H31A	0.9900
C5—C6	1.526 (3)	C31—H31B	0.9900
C5—H5A	0.9900	C32—C33	1.411 (3)
C5—H5B	0.9900	C32—C40	1.419 (3)
C6—C7	1.532 (3)	C32—Zr1	2.5539 (19)
C6—H6A	0.9900	C33—C34	1.417 (3)
C6—H6B	0.9900	C33—Zr1	2.4834 (19)
C7—C8	1.504 (2)	C33—H33	0.9500
C7—H7A	0.9900	C34—C35	1.406 (3)
C7—H7B	0.9900	C34—Zr1	2.5463 (19)
C8—C9	1.416 (3)	C34—H34	0.9500
C8—Zr2	2.5984 (18)	C35—C40	1.423 (3)
C9—C10	1.501 (3)	C35—C36	1.499 (3)
C9—Zr2	2.5428 (18)	C35—Zr1	2.6491 (18)
C10—C11	1.536 (3)	C36—C37	1.520 (3)
C10—H10A	0.9900	C36—H36A	0.9900
C10—H10B	0.9900	C36—H36B	0.9900
C11—C12	1.501 (3)	C37—C38	1.518 (3)
C11—H11A	0.9900	C37—H37A	0.9900
C11—H11B	0.9900	C37—H37B	0.9900
C12—C13	1.411 (3)	C38—C39	1.534 (3)
C12—C20	1.417 (3)	C38—H38A	0.9900
C12—Zr2	2.5522 (17)	C38—H38B	0.9900
C13—C14	1.418 (3)	C39—C40	1.503 (3)
C13—Zr2	2.4820 (17)	C39—H39A	0.9900
C13—H13	0.9500	C39—H39B	0.9900
C14—C15	1.404 (3)	C40—Zr1	2.6105 (18)
C14—Zr2	2.5396 (18)	C41—C46	1.382 (3)
C14—H14	0.9500	C41—C42	1.386 (3)
C15—C20	1.419 (3)	C41—C47	1.494 (3)
C15—C16	1.498 (3)	C42—C43	1.380 (4)
C15—Zr2	2.6339 (18)	C42—H42	0.9500
C16—C17	1.517 (3)	C43—C44	1.365 (5)
C16—H16A	0.9900	C43—H43	0.9500
C16—H16B	0.9900	C44—C45	1.373 (5)
C17—C18	1.506 (3)	C44—H44	0.9500
C17—H17A	0.9900	C45—C46	1.392 (4)
C17—H17B	0.9900	C45—H45	0.9500
C18—C19	1.555 (3)	C46—H46	0.9500
C18—H18A	0.9900	C47—H47A	0.9800
C18—H18B	0.9900	C47—H47B	0.9800
C19—C20	1.508 (3)	C47—H47C	0.9800
C19—H19A	0.9900	S1—Zr1	2.4744 (5)
C19—H19B	0.9900	S1—Zr2	2.4757 (5)
C20—Zr2	2.5956 (18)	S2—Zr1	2.4648 (5)
C21—C22	1.413 (3)	S2—Zr2	2.4684 (5)
			
C26A—C25A—C24	117.7 (3)	C32—C31—H31A	109.4
C26A—C25A—H25A	107.9	C30—C31—H31A	109.4
C24—C25A—H25A	107.9	C32—C31—H31B	109.4
C26A—C25A—H25B	107.9	C30—C31—H31B	109.4
C24—C25A—H25B	107.9	H31A—C31—H31B	108.0
H25A—C25A—H25B	107.2	C33—C32—C40	107.52 (17)
C27—C26A—C25A	105.2 (3)	C33—C32—C31	126.76 (18)
C27—C26A—H26A	110.7	C40—C32—C31	125.72 (18)
C25A—C26A—H26A	110.7	C33—C32—Zr1	71.01 (11)
C27—C26A—H26B	110.7	C40—C32—Zr1	76.26 (11)
C25A—C26A—H26B	110.7	C31—C32—Zr1	118.85 (13)
H26A—C26A—H26B	108.8	C32—C33—C34	108.38 (17)
C24—C25B—C26B	102.7 (3)	C32—C33—Zr1	76.51 (11)
C24—C25B—H25C	111.2	C34—C33—Zr1	76.09 (11)
C26B—C25B—H25C	111.2	C32—C33—H33	125.8
C24—C25B—H25D	111.2	C34—C33—H33	125.8
C26B—C25B—H25D	111.2	Zr1—C33—H33	113.9
H25C—C25B—H25D	109.1	C35—C34—C33	108.15 (16)
C25B—C26B—C27	117.7 (4)	C35—C34—Zr1	78.37 (11)
C25B—C26B—H26C	107.9	C33—C34—Zr1	71.20 (11)
C27—C26B—H26C	107.9	C35—C34—H34	125.9
C25B—C26B—H26D	107.9	C33—C34—H34	125.9
C27—C26B—H26D	107.9	Zr1—C34—H34	116.5
H26C—C26B—H26D	107.2	C34—C35—C40	107.77 (16)
C9—C1—C2	108.54 (17)	C34—C35—C36	129.39 (17)
C9—C1—Zr2	76.50 (11)	C40—C35—C36	122.50 (17)
C2—C1—Zr2	76.81 (11)	C34—C35—Zr1	70.30 (10)
C9—C1—H1	125.7	C40—C35—Zr1	72.81 (10)
C2—C1—H1	125.7	C36—C35—Zr1	127.59 (12)
Zr2—C1—H1	113.3	C35—C36—C37	110.20 (16)
C3—C2—C1	108.07 (16)	C35—C36—H36A	109.6
C3—C2—Zr2	78.39 (11)	C37—C36—H36A	109.6
C1—C2—Zr2	70.44 (10)	C35—C36—H36B	109.6
C3—C2—H2	126.0	C37—C36—H36B	109.6
C1—C2—H2	126.0	H36A—C36—H36B	108.1
Zr2—C2—H2	117.2	C38—C37—C36	110.35 (17)
C2—C3—C8	107.62 (16)	C38—C37—H37A	109.6
C2—C3—C4	129.37 (17)	C36—C37—H37A	109.6
C8—C3—C4	122.64 (17)	C38—C37—H37B	109.6
C2—C3—Zr2	70.36 (11)	C36—C37—H37B	109.6
C8—C3—Zr2	72.11 (10)	H37A—C37—H37B	108.1
C4—C3—Zr2	128.37 (13)	C37—C38—C39	111.68 (18)
C3—C4—C5	109.63 (16)	C37—C38—H38A	109.3
C3—C4—H4A	109.7	C39—C38—H38A	109.3
C5—C4—H4A	109.7	C37—C38—H38B	109.3
C3—C4—H4B	109.7	C39—C38—H38B	109.3
C5—C4—H4B	109.7	H38A—C38—H38B	107.9
H4A—C4—H4B	108.2	C40—C39—C38	111.53 (16)
C4—C5—C6	110.04 (17)	C40—C39—H39A	109.3
C4—C5—H5A	109.7	C38—C39—H39A	109.3
C6—C5—H5A	109.7	C40—C39—H39B	109.3
C4—C5—H5B	109.7	C38—C39—H39B	109.3
C6—C5—H5B	109.7	H39A—C39—H39B	108.0
H5A—C5—H5B	108.2	C32—C40—C35	108.08 (16)
C5—C6—C7	112.21 (17)	C32—C40—C39	128.66 (17)
C5—C6—H6A	109.2	C35—C40—C39	122.41 (17)
C7—C6—H6A	109.2	C32—C40—Zr1	71.87 (10)
C5—C6—H6B	109.2	C35—C40—Zr1	75.81 (10)
C7—C6—H6B	109.2	C39—C40—Zr1	126.58 (13)
H6A—C6—H6B	107.9	C46—C41—C42	118.5 (2)
C8—C7—C6	111.71 (16)	C46—C41—C47	121.1 (2)
C8—C7—H7A	109.3	C42—C41—C47	120.4 (2)
C6—C7—H7A	109.3	C43—C42—C41	120.9 (3)
C8—C7—H7B	109.3	C43—C42—H42	119.5
C6—C7—H7B	109.3	C41—C42—H42	119.5
H7A—C7—H7B	107.9	C44—C43—C42	120.4 (3)
C9—C8—C3	108.38 (16)	C44—C43—H43	119.8
C9—C8—C7	128.31 (16)	C42—C43—H43	119.8
C3—C8—C7	122.55 (17)	C43—C44—C45	119.7 (3)
C9—C8—Zr2	71.87 (10)	C43—C44—H44	120.2
C3—C8—Zr2	76.44 (10)	C45—C44—H44	120.2
C7—C8—Zr2	125.69 (12)	C44—C45—C46	120.5 (3)
C1—C9—C8	107.25 (16)	C44—C45—H45	119.8
C1—C9—C10	126.46 (17)	C46—C45—H45	119.8
C8—C9—C10	126.29 (17)	C41—C46—C45	120.1 (2)
C1—C9—Zr2	70.82 (10)	C41—C46—H46	120.0
C8—C9—Zr2	76.19 (10)	C45—C46—H46	120.0
C10—C9—Zr2	119.11 (12)	C41—C47—H47A	109.5
C9—C10—C11	112.09 (15)	C41—C47—H47B	109.5
C9—C10—H10A	109.2	H47A—C47—H47B	109.5
C11—C10—H10A	109.2	C41—C47—H47C	109.5
C9—C10—H10B	109.2	H47A—C47—H47C	109.5
C11—C10—H10B	109.2	H47B—C47—H47C	109.5
H10A—C10—H10B	107.9	Zr1—S1—Zr2	90.427 (15)
C12—C11—C10	110.96 (15)	Zr1—S2—Zr2	90.824 (15)
C12—C11—H11A	109.4	S2—Zr1—C21	129.41 (5)
C10—C11—H11A	109.4	S2—Zr1—S1	89.383 (15)
C12—C11—H11B	109.4	C21—Zr1—S1	93.11 (5)
C10—C11—H11B	109.4	S2—Zr1—C33	93.72 (5)
H11A—C11—H11B	108.0	C21—Zr1—C33	120.12 (7)
C13—C12—C20	107.18 (16)	S1—Zr1—C33	131.65 (5)
C13—C12—C11	126.39 (17)	S2—Zr1—C22	97.31 (5)
C20—C12—C11	126.43 (17)	C21—Zr1—C22	32.76 (7)
C13—C12—Zr2	71.00 (10)	S1—Zr1—C22	86.40 (5)
C20—C12—Zr2	75.72 (10)	C33—Zr1—C22	140.52 (6)
C11—C12—Zr2	118.65 (12)	S2—Zr1—C34	86.16 (4)
C12—C13—C14	108.48 (16)	C21—Zr1—C34	142.41 (7)
C12—C13—Zr2	76.48 (10)	S1—Zr1—C34	99.79 (4)
C14—C13—Zr2	75.84 (10)	C33—Zr1—C34	32.71 (6)
C12—C13—H13	125.8	C22—Zr1—C34	172.97 (6)
C14—C13—H13	125.8	S2—Zr1—C29	127.41 (5)
Zr2—C13—H13	114.2	C21—Zr1—C29	32.67 (6)
C15—C14—C13	108.06 (16)	S1—Zr1—C29	124.97 (5)
C15—C14—Zr2	77.99 (11)	C33—Zr1—C29	89.95 (6)
C13—C14—Zr2	71.38 (10)	C22—Zr1—C29	53.71 (6)
C15—C14—H14	126.0	C34—Zr1—C29	119.41 (6)
C13—C14—H14	126.0	S2—Zr1—C32	125.62 (5)
Zr2—C14—H14	116.7	C21—Zr1—C32	91.02 (7)
C14—C15—C20	107.71 (16)	S1—Zr1—C32	127.88 (4)
C14—C15—C16	128.51 (17)	C33—Zr1—C32	32.49 (6)
C20—C15—C16	123.43 (17)	C22—Zr1—C32	119.96 (6)
C14—C15—Zr2	70.58 (10)	C34—Zr1—C32	53.45 (6)
C20—C15—Zr2	72.77 (10)	C29—Zr1—C32	66.57 (6)
C16—C15—Zr2	127.54 (12)	S2—Zr1—C28	95.81 (4)
C15—C16—C17	111.01 (17)	C21—Zr1—C28	53.08 (6)
C15—C16—H16A	109.4	S1—Zr1—C28	139.01 (5)
C17—C16—H16A	109.4	C33—Zr1—C28	88.67 (6)
C15—C16—H16B	109.4	C22—Zr1—C28	52.62 (6)
C17—C16—H16B	109.4	C34—Zr1—C28	121.08 (6)
H16A—C16—H16B	108.0	C29—Zr1—C28	31.78 (6)
C18—C17—C16	111.10 (19)	C32—Zr1—C28	80.41 (6)
C18—C17—H17A	109.4	S2—Zr1—C40	138.74 (4)
C16—C17—H17A	109.4	C21—Zr1—C40	91.20 (7)
C18—C17—H17B	109.4	S1—Zr1—C40	96.07 (4)
C16—C17—H17B	109.4	C33—Zr1—C40	53.16 (6)
H17A—C17—H17B	108.0	C22—Zr1—C40	123.79 (6)
C17—C18—C19	112.46 (19)	C34—Zr1—C40	52.59 (6)
C17—C18—H18A	109.1	C29—Zr1—C40	81.43 (6)
C19—C18—H18A	109.1	C32—Zr1—C40	31.87 (6)
C17—C18—H18B	109.1	C28—Zr1—C40	105.89 (6)
C19—C18—H18B	109.1	S2—Zr1—C23	79.60 (4)
H18A—C18—H18B	107.8	C21—Zr1—C23	52.77 (6)
C20—C19—C18	110.16 (17)	S1—Zr1—C23	111.74 (5)
C20—C19—H19A	109.6	C33—Zr1—C23	116.28 (6)
C18—C19—H19A	109.6	C22—Zr1—C23	31.33 (6)
C20—C19—H19B	109.6	C34—Zr1—C23	145.01 (6)
C18—C19—H19B	109.6	C29—Zr1—C23	52.55 (6)
H19A—C19—H19B	108.1	C32—Zr1—C23	111.73 (6)
C12—C20—C15	108.46 (16)	C28—Zr1—C23	31.41 (6)
C12—C20—C19	128.45 (17)	C40—Zr1—C23	133.87 (6)
C15—C20—C19	122.33 (17)	S2—Zr1—C35	110.94 (4)
C12—C20—Zr2	72.35 (10)	C21—Zr1—C35	119.37 (6)
C15—C20—Zr2	75.75 (10)	S1—Zr1—C35	81.18 (4)
C19—C20—Zr2	125.86 (13)	C33—Zr1—C35	52.79 (6)
C22—C21—C29	108.69 (18)	C22—Zr1—C35	148.84 (6)
C22—C21—Zr1	76.17 (11)	C34—Zr1—C35	31.32 (6)
C29—C21—Zr1	76.67 (11)	C29—Zr1—C35	112.61 (6)
C22—C21—H21	125.7	C32—Zr1—C35	52.44 (6)
C29—C21—H21	125.7	C28—Zr1—C35	132.85 (6)
Zr1—C21—H21	113.8	C40—Zr1—C35	31.38 (6)
C23—C22—C21	107.98 (18)	C23—Zr1—C35	163.98 (6)
C23—C22—Zr1	78.49 (11)	S2—Zr2—C1	93.14 (5)
C21—C22—Zr1	71.07 (11)	S2—Zr2—S1	89.273 (15)
C23—C22—H22	126.0	C1—Zr2—S1	132.29 (5)
C21—C22—H22	126.0	S2—Zr2—C13	130.15 (5)
Zr1—C22—H22	116.5	C1—Zr2—C13	119.74 (6)
C22—C23—C28	107.79 (18)	S1—Zr2—C13	93.18 (4)
C22—C23—C24	129.78 (19)	S2—Zr2—C14	97.95 (4)
C28—C23—C24	122.13 (19)	C1—Zr2—C14	139.48 (6)
C22—C23—Zr1	70.18 (11)	S1—Zr2—C14	86.88 (4)
C28—C23—Zr1	72.99 (11)	C13—Zr2—C14	32.78 (6)
C24—C23—Zr1	127.26 (13)	S2—Zr2—C9	125.51 (4)
C23—C24—C25B	114.2 (3)	C1—Zr2—C9	32.69 (6)
C23—C24—C25A	109.5 (2)	S1—Zr2—C9	127.22 (4)
C23—C24—H24A	109.8	C13—Zr2—C9	91.07 (6)
C25B—C24—H24A	127.8	C14—Zr2—C9	119.98 (6)
C25A—C24—H24A	109.8	S2—Zr2—C2	84.28 (4)
C23—C24—H24B	109.8	C1—Zr2—C2	32.75 (6)
C25B—C24—H24B	82.0	S1—Zr2—C2	100.62 (5)
C25A—C24—H24B	109.8	C13—Zr2—C2	143.25 (6)
H24A—C24—H24B	108.2	C14—Zr2—C2	172.23 (6)
C23—C24—H24C	108.7	C9—Zr2—C2	53.61 (6)
C25B—C24—H24C	108.7	S2—Zr2—C12	128.41 (4)
C25A—C24—H24C	84.7	C1—Zr2—C12	89.44 (6)
H24B—C24—H24C	130.6	S1—Zr2—C12	124.79 (4)
C23—C24—H24D	108.7	C13—Zr2—C12	32.52 (6)
C25B—C24—H24D	108.7	C14—Zr2—C12	53.60 (6)
C25A—C24—H24D	133.0	C9—Zr2—C12	66.68 (6)
H24A—C24—H24D	81.3	C2—Zr2—C12	119.37 (6)
H24C—C24—H24D	107.6	S2—Zr2—C20	96.68 (4)
C28—C27—C26A	113.3 (2)	C1—Zr2—C20	87.44 (6)
C28—C27—C26B	106.8 (3)	S1—Zr2—C20	139.56 (4)
C28—C27—H27A	108.9	C13—Zr2—C20	53.20 (6)
C26A—C27—H27A	108.9	C14—Zr2—C20	52.70 (6)
C26B—C27—H27A	136.6	C9—Zr2—C20	80.33 (6)
C28—C27—H27B	108.9	C2—Zr2—C20	119.74 (6)
C26A—C27—H27B	108.9	C12—Zr2—C20	31.94 (6)
C26B—C27—H27B	82.6	S2—Zr2—C8	136.81 (4)
H27A—C27—H27B	107.7	C1—Zr2—C8	53.32 (6)
C28—C27—H27C	110.4	S1—Zr2—C8	95.29 (4)
C26A—C27—H27C	79.0	C13—Zr2—C8	92.54 (6)
C26B—C27—H27C	110.4	C14—Zr2—C8	125.15 (6)
H27B—C27—H27C	132.2	C9—Zr2—C8	31.94 (6)
C28—C27—H27D	110.4	C2—Zr2—C8	52.64 (6)
C26A—C27—H27D	129.3	C12—Zr2—C8	82.58 (6)
C26B—C27—H27D	110.4	C20—Zr2—C8	106.67 (6)
H27A—C27—H27D	79.1	S2—Zr2—C15	80.34 (4)
H27C—C27—H27D	108.6	C1—Zr2—C15	114.95 (6)
C29—C28—C23	108.41 (17)	S1—Zr2—C15	112.42 (4)
C29—C28—C27	128.33 (18)	C13—Zr2—C15	52.93 (6)
C23—C28—C27	122.78 (18)	C14—Zr2—C15	31.43 (6)
C29—C28—Zr1	71.76 (10)	C9—Zr2—C15	111.73 (6)
C23—C28—Zr1	75.61 (10)	C2—Zr2—C15	143.12 (6)
C27—C28—Zr1	124.98 (13)	C12—Zr2—C15	52.64 (6)
C28—C29—C21	107.05 (17)	C20—Zr2—C15	31.47 (6)
C28—C29—C30	125.68 (18)	C8—Zr2—C15	135.04 (6)
C21—C29—C30	127.27 (19)	S2—Zr2—C3	108.38 (4)
C28—C29—Zr1	76.46 (10)	C1—Zr2—C3	52.82 (6)
C21—C29—Zr1	70.66 (10)	S1—Zr2—C3	81.31 (4)
C30—C29—Zr1	118.71 (13)	C13—Zr2—C3	121.21 (6)
C29—C30—C31	111.51 (16)	C14—Zr2—C3	150.86 (6)
C29—C30—H30A	109.3	C9—Zr2—C3	52.56 (6)
C31—C30—H30A	109.3	C2—Zr2—C3	31.24 (6)
C29—C30—H30B	109.3	C12—Zr2—C3	113.69 (6)
C31—C30—H30B	109.3	C20—Zr2—C3	132.85 (6)
H30A—C30—H30B	108.0	C8—Zr2—C3	31.45 (5)
C32—C31—C30	111.19 (16)	C15—Zr2—C3	164.28 (6)
